# Genome wide SNP comparative analysis between EGFR and KRAS mutated NSCLC and characterization of two models of oncogenic cooperation in non-small cell lung carcinoma

**DOI:** 10.1186/1755-8794-1-25

**Published:** 2008-06-12

**Authors:** Hélène Blons, Karine Pallier, Delphine Le Corre, Claire Danel, Maxime Tremblay-Gravel, Claude Houdayer, Elizabeth Fabre-Guillevin, Marc Riquet, Philippe Dessen, Pierre Laurent-Puig

**Affiliations:** 1 UMR-S775, INSERM, Paris, F-75006, France; 2 Université Paris Descartes, Paris, F-75006, France; 3 Department of Biology, Hôpital Européen Georges Pompidou, AP-HP, Paris, F-75015, France; 4 Department of Pathology, Hôpital Européen Georges Pompidou, AP-HP, Paris, F-75015, France; 5 Department of Molecular Oncology, Institut Curie, Paris, F-75005, France; 6 Department of Oncology, Hôpital Européen Georges Pompidou, AP-HP, Paris, F-75015, France; 7 Department of Thoracic Surgery, Hôpital Européen Georges Pompidou, AP-HP, Paris, F-75015, France; 8 Department of Informatics, Institut Gustave Roussy, Villejuif, F-94905, France

## Abstract

**Background:**

Lung cancer with EGFR mutation was shown to be a specific clinical entity. In order to better understand the biology behind this disease we used a genome wide characterization of loss of heterozygosity and amplification by Single Nucleotide Polymorphism (SNP) Array analysis to point out chromosome segments linked to *EGFR *mutations. To do so, we compared genetic profiles between *EGFR *mutated adenocarcinomas (ADC) and *KRAS *mutated ADC from 24 women with localized lung cancer.

**Results:**

Patterns of alterations were different between *EGFR *and *KRAS *mutated tumors and specific chromosomes alterations were linked to the *EGFR *mutated group. Indeed chromosome regions 14q21.3 (p = 0.027), 7p21.3-p21.2 (p = 0.032), 7p21.3 (p = 0.042) and 7p21.2-7p15.3 (p = 0.043) were found significantly amplified in EGFR mutated tumors. Within those regions 3 genes are of special interest *ITGB8*, *HDAC9 *and *TWIST1*. Moreover, homozygous deletions at *CDKN2A *and LOH at *RB1 *were identified in *EGFR *mutated tumors. We therefore tested the existence of a link between EGFR mutation, CDKN2A homozygous deletion and cyclin amplification in a larger series of tumors. Indeed, in a series of non-small-cell lung carcinoma (n = 98) we showed that homozygous deletions at *CDKN2A *were linked to *EGFR *mutations and absence of smoking whereas cyclin amplifications (*CCNE1 *and *CCND1*) were associated to *TP53 *mutations and smoking habit.

**Conclusion:**

All together, our results show that genome wide patterns of alteration differ between *EGFR *and *KRAS *mutated lung ADC, describe two models of oncogenic cooperation involving either *EGFR *mutation and *CDKN2A *deletion or cyclin amplification and *TP53 *inactivating mutations and identified new chromosome regions at 7p and 14q associated to EGFR mutations in lung cancer.

## Background

Lung cancer is the leading cause of cancer-related deaths in the western world [1]. Non-small cell lung cancer (NSCLC) accounts for approximately 85% of the cases and represents a heterogeneous group mainly consisting of adenocarcinoma (ADC), large cell carcinoma (LCC) and squamous cell carcinoma (SCC). The incidence of subtypes has changed in the last decades with increasing incidence of ADC. Moreover, while smoking remains the major risk factor for lung cancer, a subgroup of patients develop lung ADC without smoking history. It is not clear whether lung cancer in non-smokers is increasing in western countries but it is obvious that it has particular clinical and biological features. Population based studies showed that lung cancer in non-smokers occurs preferentially in women with almost 20% of non-smoker lung cancer diagnosed in women versus 2.5% in men [2]. Different studies have shown that genetic abnormalities can be specifically identified in cancer from non-smokers. Indeed we and others showed that *KRAS *mutations were linked to tobacco consumption whereas EGF receptor *(EGFR) *mutations were found in non smokers [3-5]. The development of EGFR targeted therapies demonstrated that patients with major clinical response were those that had never smoked, had ADC with bronchioloalveolar component, were women and had *EGFR *mutations [6-8]. The biology underlying the pathogenesis of the disease may be different from that of smokers and risk factors have not been clearly identified although environmental etiologies are suspected especially in Asians [9]. Transformation of a normal phenotype into a malignant phenotype requires accumulation of multiple genetic and-or epigenetic changes resulting in growth advantage.

The genetic alteration proved to be linked with ADC from non-smokers is the presence of *EGFR *activating mutations. In order to improve our knowledge of lung cancer biology in non-smokers, one of the first questions to answer is: what are the molecular alterations associated to *EGFR *mutations in lung cancer?

Lung cancer develops as a result of multiple genetic alterations. Loss of heterozygosity (LOH) and gains of chromosome segments are common mechanisms of disease progression. Recently, high-density oligonucleotide-based single polymorphism have been used to quantify chromosome copy number and has been proved to be efficient [10-12]. In an attempt to identify genetic alterations associated with *EGFR *mutations, we used genome wide SNP assay covering 50000 SNP loci to screen for regions of allelic imbalance (amplified or LOH regions) in a panel of 13 *EGFR *mutated ADC and 11 non-*EGFR *mutated ADC.

Then, in a second part, we focused on cell cycle genes that were found to be differentially involved between groups and screened a large series of 98 NSCLC for genetic alterations at *CCND1*, *CCNE1*, *CDKN2A *and *RB1*. Alterations were studied according to other known mutations (*EGFR*, *ERBB2*, *BRAF*, *KRAS*, *TP53 *and *STK11*). This work led to the characterization of two different models of oncogenic cooperation one linked to smoking and the other not. Moreover, we identified four chromosome regions at 14q and 7p specifically amplified in EGFR mutated ADC.

## Patients and methods

Patients with primary lung cancers were enrolled in this study according to French laws and have been previously described [4]. Briefly, patients had surgery for non-small cell lung cancer, no neoadjuvant treatment and were managed to the Georges Pompidou European Hospital in Paris, France from 2003 to 2004. All tumors but numbers 134, 135, 177 and 246 had been characterized for mutations in *EGFR*, *KRAS*, *BRAF*, *ERBB2*, *ERBB3*, *PIK3CA*, *TP53 *and *STK11 *[4]. *STK11 *mutations have not been previously published. Patient characteristics are summarized in Table 3. DNAs were extracted after pulverization in liquid nitrogen and protein kinase digestion using Qiamp tissue kit (Qiagen, Les Ulis, France). Twenty-four DNAs (13 with classic *EGFR *mutations, 11 without) and 6 non-tumor DNAs were selected for SNP array analysis. All tumors selected were from women.

### Single Nucleotide Polymorphism Array Analysis

Genechip^® ^Mapping 50K-Xba array was used for this analysis. Preparation of DNA targets, labelling, hybridization, washing, staining and scanning was done according to the manufacturer's instructions (Affymetrix, UK) by PartnerChip (Evry, France).

Data were analyzed using Copy Number Analyser for Affymetrix Gene Chip Mapping (CNAG 2.0) software [13]. We selected randomly 18 independent subjects from Mapping 100k HapMap Trio Dataset provided by affymetrix. Indeed, this software/algorithm uses a set of normal reference individuals and do not require the use of a paired normal sample to perform the analysis.

Data from CNAG were export in aCGH sotfware (R package) that was used for plot performance and statistical comparison of *EGFR *mutated and non-mutated tumors.

### Quantitative PCR experiments

Validation of homozygous deletions (*CDKN2A*) and regions with focal amplification (*CCND1 *and *CCNE1*) was done on the 24 tumors screened by SNP array and extended to a total of 98 NSCLC (Table 2). Human serum albumin (*HSA*) was used as the reference gene. DNA concentrations were determined using ND-1000 spectrophotometer Nanodrop technology and were normalized to 12,5ng/ul. Real time quantitative PCR using TaqMan probes was performed using an ABI Prism 7900 sequence detection system (Applied Biosystems, Courtaboeuf, France) with the software program SDS 2.0 (Applied Biosystems). Each assay was run on a 384 plate, tumor DNAs, normal controls (n = 6) and no template controls were run in triplicates for *CCND1*, *CCNE1*, *CDKN2A *and *HSA*. Primers and probes were described elsewhere [14]. Homozygous deletions at *DCC*, *DSG2 *and *DSC3 *as well as copy number changes of the *HDAC9*, *TWIST1 *and *ITGB8 *region were validated by real time PCR for the 24 tumors screened by SNP array. Primers and probes where designed with Primer Express 2.0 software program (Applied Biosystems) (see Additional file 1). All primers were purchased from Operon (Cologne, Germany) and probes from Applied Biosystems.

The PCR mix consisted of ABsolute™ QPCR MIX 1× (ABgene, Courtaboeuf, France), primers 300 nM, probe 200 nM, H_2_0 and 25 ng of DNA template in a final volume of 10 ul. Cycling condition were denaturation 95°C for 15 min and 40 cycles of 95°C, 15 sec followed by 60°C, 15 sec. Quantification was done by normalizing the results to those of *HSA*. The normalized amount of gene in tumor samples was determined by designating the average of ΔCt of 6 non-tumor tissues as calibrator. 2 × 2^-ΔΔCt ^represented an estimation of the number of gene copy in tumor tissues.

The cutoff value was 2 × 2^-ΔΔCt ^≤ 0.6 for homozygous deletion and 2 × 2^-ΔΔCt ^≥ 4 for amplifications.

### *STK11 *Mutations screening

Exons 1 to 9 were screened by direct sequencing. Primers used for the amplification and sequencing of each exon and intron-exon junctions and PCR conditions are available upon request.

### Statistical analysis

Fractional allelic loss (FAL) and fractional allelic amplification (FAA) were calculated for each tumor as the number of chromosome arms with either loss of heterozygosity or amplifications divided by the number of chromosome arms tested (41). Mean FAL and FAA were compared using student T test. Qualitative variables were compared using chi square test or Fisher exact test when necessary. All tests were performed using STATA 7.0 (StataCorp LP, College Station, TX) aCGH software was used to test the existence of meaningful differences between focal chromosome alterations in *EGFR *mutated and non-mutated tumors. False discovery rate test (FDR) has been used to assess p values. FDR represents the expected percentage of false positive among the claimed positive and estimates global error for multiple testing situations. Therefore p values were adjusted to the number of tests performed.

## Results

### Array global analysis

Mapping genome wide chromosomal alterations in *EGFR *mutated lung ADC (n = 13) versus non-mutated ones (n = 11) had two different objectives. First, a global comparison of allelic imbalances and second, a targeted analysis of specific loci to point out genes implicated in the oncogenesis of one subtype or the other. It is to note that, in an effort to homogenize both groups, non-EGFR mutated ADC are all *KRAS *mutated. Fractional allelic loss (FAL) and fractional allelic amplification (FAA) were calculated as the number of chromosome arm with LOH or amplified loci divided by 41 chromosome arms.

**Figure 1:**
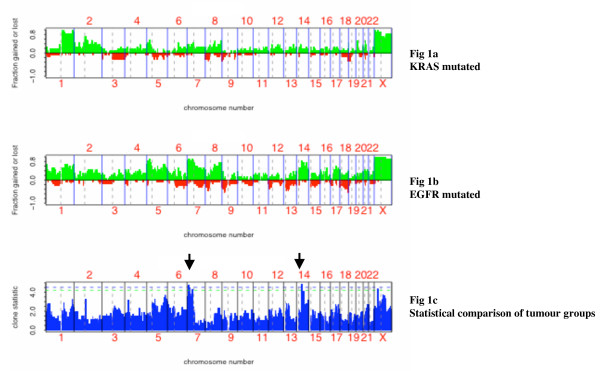
**DNA copy number alterations by SNP array analysis. ** Figure 1 represents the fraction of the samples with copy number amplification of at least  three copies (green) and copy number reduction (red) across all chromosome SNPs; in the  EGFR non-mutated/KRAS mutated group (A) and the EGFR mutated/ KRAS non-mutated  group (B). (C) Statistical comparison of both groups showing regions of amplification  20  statistically linked to EGFR mutated tumors (black arrows). False discovery rate (FDR) has  been used to estimate global error for multiple testing situations.

Mean FALs were equal to 0.16 (± 0.08) and 0.09 (± 0.05), (p = 0.024) in the *EGFR *and the *KRAS *mutated group respectively. Chromosome arms that were involved in LOH (copy number ≤ 1) in more than 25% of tumors were, in the *EGFR *mutated group 6q, 7q, 8p, 9p, 10q, 12p, 12q, 13q, 15q and 18q, and in the *KRAS *mutated group 3q, 5q, 8p and 19p. A single copy of chromosome 9, 13, 15, 18 and 22 was found in at least one *EGFR *tumor, no monosomy was found in the *KRAS *group (Figure 1a and 1b).

Concerning amplifications, mean FAAs were equal to 0.50 (± 0.19) and 0.33 (± 0.24), (p = 0.039) in the *EGFR *versus the *KRAS *mutated group. Chromosome that had amplified regions (copy number ≥ 3) at high rates (> 50% of tumors) were 1p-q, 2p, 3q, 5q, 7p-q, 8q, 14q, 17q, 21q and Xp-q in the *EGFR *mutated group and 1q, 2p-q, 5p, 8q and Xp-q in the *KRAS *mutated group (Figure 1a and 1b).

### Array targeted analysis

Differential analysis between *EGFR *mutated and *KRAS *mutated tumors using aCGH package [15] showed that one region at 14q (p = 0.027) and three regions at 7p (p = 0.032, p = 0.042, p = 0.043) were statistically more frequently amplified in the *EGFR *group (Figure 1c). Detailed statistic analysis is shown as supplementary data (see Additional file 2). Genes located in these regions are given in Table 1. The gene located at 14q is a MAM domain protein. The MAM domain is present in many cell surface proteins and is thought to be involved in cell-cell adhesion, protein-protein interactions, and signal transduction, whether this protein could be linked to carcinogenesis remain to be studied [16]. Of the ones located at 7p21.1, it is to note that *HDAC9, TWIST1 *and *ITGB8 *are potential targets. Gene copy numbers at 7p21.1 were validated by quantitative PCR using probes *HDAC9 *and *ITGB8 *in the 24 tumors and 7 cell lines were tested for copy number changes using same probes (Calu6-H460-A549-H1299-H1650-H1975-H358), both cell lines with *EGFR *mutation were found amplified (H1650-H1975) versus one out of five without EGFR mutations (H358). *EGFR *is located at 7p11.2, among tumors with *EGFR *mutations, one has no copy number alteration at 7p, 9 showed concomitant 7p11.2 and 7p21.1 amplifications (>3) and 3 had a localized amplification, 2 at 7p21.1 and 1 at 7p11.2. EGFR amplifications were not statistically related with EGFR mutation but 5/13 in the EGFR group versus 1/11 in the KRAS group had estimated copy number > 4.

**Table 1:** Shows regions of focal amplifications analyzed by Xba1 50000 SNPs array (Affymetrix) that  are significantly linked to EGFR mutated tumors.

**Base position start**	**Base position end**	**Chromosome Band**	**Genes within the region**	**NAME**	**Known functions**
46489396	47088012	14q21.3	MAMDC1	MAM DOMAIN CONTAINING1	MAM domain containing protein
11727815	13456320	7p21.3-7p21.2	TMEM106B	Hypothetical transmembrane protein	unknown
			AK027618	Weakly similar to neurogenic locus notch3 protein	unknown
			AK075525	Weakly similar to UROMODULIN PRECURSOR	unknown
			BC075797	cDNA clone MGC:87550	unknown
			CR592342	cDNA clone MGC:87550	
			SCIN	SCINDERIN	Organization of microfilament network
			ARL4	ADP-ribosylation factor-like 4A	ADP-ribosylation factor family of GTP-binding protein
11120441	11377580	7p21.3	PHF14	HD finger protein 14 isoform 1	unknown
			BC040327	Homo sapiens cDNA clone IMAGE:4830466	unknown
18006698	20386036	7p21.1-7p15.3	PRPS1L1	PRPS1L1	Phosphoribosyl pyrophosphate synthetase 1-like
			HDAC9	Histone deacetylase 9 isoform 4	Histone acetylation/deacetylation alters chromosome structure and affects transcription factor access to DNA
			TWIST1	TWIST1	The protein encoded by this gene is a bHLH transcription factor. Basic helix-loop-helix (bHLH) transcription factors have been implicated in cell lineage determination and differentiation.
			FERD3L	Nephew of atonal 3	N-TWIST basic (helix-loop-helix protein)
			TWISTNB	TWIST neighbor	DNA-directed RNA polymerase I subunit RPA43-transcription
			MGC42090	Hypothetical protein LOC256130	unknown
			7A5	Putative binding protein 7a5	unknown
			ITGB8	integrin, beta 8 precursor	This gene is a member of the integrin beta chain family. Integrin complexes mediate cell-cell and cell-extracellular matrix interactions and this complex plays a role in human airway epithelial proliferation.

Regardless of statistical difference between the two groups, focal amplifications and homozygous deletion are of special interest as they may indicate oncogenes or tumor suppressors. Recurrent regions of deletions and focal amplifications were defined as segments of at least 5 SNP loci in more than 2 tumors.

A total of 58 regions with focal amplification were identified. Among them 22 regions were found exclusively in *EGFR *mutated tumors versus 2 in *KRAS *mutated tumors. Genes potentially involved in carcinogenesis located in these regions are listed as supplementary material (see Additional file 3), *AFF3*, *LAF4*, *LRRC1*, *SNW1*, *FGF7*, *PPL *and *CTAGE1 *for *EGFR *mutated tumors and *MYST3*, *IKBKB*, *DKK4 *for *KRAS *mutated tumors. Regions of amplifications common to both groups delineated by the SNP array included *BCL9*, *FGF10*, *IL31RA*, *PLK2*, *WISP1*, *HTERT*, *CCND3*, *EFGR*, *MYC*.

Homozygous deletions were identified at chromosome regions 2q36.3, 9p21.1, 12q13.13, 18q12.1 and 18q21.2. The 9p21.1 locus contains *CDKN2A *and the 18q21.1 contains *DCC*, both are well known tumor suppressors. Genes in the 12q13.13 and 2q36.3 regions have not been linked to cancer up to now and the 18q12.1 region contains a cluster of genes coding desmosomal proteins. All homozygous deletions were found in the *EGFR *mutated group (Table 2). For the 24 tumors, quantitative PCR was ran to estimated gene copy number at *CDKN2A*, *DSG3*, *DSC2 *and *DCC *and validated the results of the SNP Array (see Additional file 4).

**Table 2:** Regions of homozygous deletions identified by SNP array in this series.

**CHR**	**CYTOBAND**	**Deletion START**	**Deletion END**	**No OF T WITHOUT EGFR MUT**	**No OF T WITH EGFR MUT**	**KNOWN GENES WITHIN REGIONS**	**GENES**		
2	2q36.3	227428860	228480608	0	2(1LOH+1HOMO)	3	RHBDD1	COL4A4	COL4A3
9	9p21.1	21701815	22685593	2 (LOH)	7(4LOH+3HOMO)	3	**CDKN2A/2B**	MTAP	DMRTA1
12	12q13.13	49778776	50467951	1(LOH)	1HOMO	3	TFCP2	POU6F1	DAZAP2
18	18q12.1	25534701	27580619	2(LOH)	3(2LOH+1HOMO)	3	CDH2	**DSC3**	**DSG2**
18	18q21.2	46939466	49883403	2(LOH)	4(3LOH+1HOMO)	2	RKHD2	**DCC**	

Homozygous  deletion were validated by real time quantitative PCR for CDKN2A, DCC, DSC3 and DSG2.

### Cell cycle related genes and lung carcinogenesis

The presence of *CDKN2A *homozygous deletion was restricted to the *EGFR *group and concerned 3 tumors out of 13 while LOH at this locus was present in 4 *EGFR *mutated tumors versus 2 non-mutated ones. Furthermore, since LOH at *RB1 *was restricted to *EGFR *mutated tumors we made the hypothesis that alterations of the G1-S check point could be different in *EGFR *mutated tumors as compared to non-mutated ones. Differences at *CDKN2A *and *RB1 *did not reach statistical significance at the array level however it deserved to be confirmed in a larger series of NSCLC and enlarge to other key regulators as cyclins. LOH at *RB1 *locus clustered to the *EGFR *group, therefore we screened the entire coding sequence and exon-intron boundaries for alterations in the subgroup of 13 *EGFR *mutated tumors. As no mutation was identified in this subgroup of tumors, *RB1 *sequencing was not done on the entire series. Then, *CDKN2A *homozygous deletions, *CCNE1 *and *CCND1 *amplifications were analyzed by real time quantitative PCR on a series of 98 NSCLC including the 24 tumors previously typed by SNP array. All tumors had been characterized for *EGFR*, *ERBB2*, *PIK3CA*, *BRAF*, *KRAS *and *STK11 *mutations. Briefly, 13 and 6 NSCLC had *CCND1 *or *CCNE1 *amplification respectively and 8 tumors had homozygous deletion at *CDKN2A *locus including the 3 previously found in array analysis (Table 3).

**Table 3:** Describes the population studied in terms of clinico-pathological features, gene mutations  (EGFR, ERBB2, PIK3CA, KRAS, BRAF and TP53) as well as gene copy number (CCND1,  CCNE1 and CDKN2A) analyzed by real time PCR.

**N°**	**Sex**	**Age at diagnosis**	**Smoker**	**TNM**	**Tumor Type**	**EGFR**	**TP53**	**KRAS**	**ERBB2**	**PIK3CA**	**STK11**	CCND (2*2-DDCt)	CCNE1 (2*2-DDCt)	CDKN2A (2*2-DDCt)
2	F	49	Yes	T2N0	BAC	0	0	0	0	0	c650insG, pFs265X	2,42	2.32	0.53
3	F	61	Yes	T3N2	ADC	0	0	0	0	0	0	2,70	2.30	0.82
4	F	42	Yes	T1N0	ADC	0	C743G>T, pR248L	c34G>T pG12C	0	0	0	1,86	3.38	1.03
5	F	80	No	T4N2	ADC	0	0	0	0	0	0	1,82	2.05	0.90
6	F	41	Yes	T2N2	LCC	0	c358A>T, pK120X	0	0	0	0	3,16	2.19	2.38
7	F	39	Yes	T2Nx	LCC	0	c796G>T, pL266X	0	0	0	c153delG, pFs63X	3,13	2.36	3.42
8	F	69	No	T2N0	ADC/BAC	0	0	0	0	0	0	2.68	2.36	0.71
9	F	65	No	T2N1	ADC	0	c452454del, FsP153G	0	0	0	0	1.92	2.44	1.17
10	F	79	No	T4N0	ADC/BAC	L858R	0	0	0	0	0	2.68	2.92	0.33
12	F	52	Yes	T2N0	ADC/BAC	0	0	c35G>A pG12D	0	0	C508C>T, pQ170X	1.71	1.43	1.05
13	F	77	No	T2N0	ADC	0	0	0	0	0	0	2.28	2.26	1.22
15	F	73	Yes	T2N0	BAC	0	0	c35G>T pG12V	0	0	0	1.75	2.16	0.72
16	F	46	Yes	T2N0	ADC	0	c614A>G, pY205C	0	0	0	0	4.59	2.66	1.90
17	F	84	No	T2N0	ADC/BAC	L858R	0	0	0	0	0	1.84	1.97	0.90
18	F	65	No	T2N0	ADC/BAC	c2241_2258del p747-753del insQ747	c880delG, FsE294S	0	0	0	0	2.22	2.51	2.18
21	F	50	Yes	T2N0	ADC/BAC	0	0	c34G>T pG12C	0	0	0	2.15	2.75	1.73
22	F	78	Yes	T2N1	LCC	0	0	0	0	0	c595G>C, pE198Q	4.64	3.05	1.23
23	F	49	Yes	T2N0	LCC	0	0	0	0	0	c1072C>Q, pF354L	2.90	2.37	0.96
24	F	65	Yes	T2N0	BAC	0	c574C>T, PQ192X	c34G>T pG12C	0	0	0	1.82	2.34	1.77
25	F	55	No	T2N0	ADC/BAC	0	0	0	0	0	0	1.84	1.81	1.51
26	F	73	No	T2N2	ADC	0	c783-2A>G, SLPICEMUT	0	c2322_2323ins12, pM774_A775ins AYVM	0	0	2.60	3.56	1.68
29	F	61	No	T2N1	ADC	L858R	0	0	0	0	0	1.98	2.06	0.29
30	F	59	No	T4N2	ADC	L858R	c258-280del, FsA86A	0	0	0	0	2.31	1.47	0.59
31	F	75	No	T2N2	ADC	c2240_2257del, p747-753del insS747	0	0	0	0	0	2.08	2.31	1.02
32	F	68	Yes	T2N0	ADC	0	c848G>C, pR283P	0	0	0	0	4.44	3.52	1.75
34	F	59	Yes	T2N2	SCC	0	0	0	0	0	0	5.62	2.21	1.31
36	F	70	No	T2N0	ADC	0	0	0	0	0	0	2.87	2.24	1.21
37	F	72	Yes	T1NO	SCC	0	0	0	0	0	0	2.17	2.00	1.62
38	F	60	Yes	T2N2	ADC	0	c747G>T, pR249S	c34G>T pG12C	0	0	0	2.41	2.48	0.81
39	F	75	No	T2N0	ADC/BAC	L858R	c189G>T, pE62X	0	0	0	0	0.91	2.04	0.92
41	F	56	No	T1NO	ADC/BAC	L858R	0	0	0	0	0	2.58	3.08	1.29
42	F	43	Yes	T2N0	ADC	0	0	c35G>T pG12V	0	0	c580G>T, pD194Y	1.84	2.49	1.01
43	F	72	No	T2N0	ADC	c2310insAGCGTGGAC p770insSVD	0	0	0	0	0	2.11	2.41	0.57
44	F	63	Yes	T2N0	LCC	0	c743G>T, pR248L	0	0	0	0	2.38	10.31	0.74
45	F	60	Yes	T2N0	ADC/BAC	0	c166G>T, pE56X	0	0	0	0	2.73	2.94	1.03
46	F	57	Yes	T2N0	ADC	0	0	0	0	0	0	2.39	2.67	0.70
48	F	49	Yes	T3N0	ADC	0	0	0	0	0	0	2.58	2.98	0.83
49	F	75	No	T2N0	BAC	c2237_2255delinsT, p746-752del insV746	0	0	0	0	0	1.77	2.20	1.58
50	F	42	Yes	T3N0	LCC	0	c814G>T, pV272L	0	0	0	0	6.26	3.13	2.40
51	F	51	Yes	n.d.	SCC	0	0	0	0	0	0	3.52	3.18	1.58
52	F	78	No	T2N2	ADC	0	0	0	0	0	0	1.77	2.13	1.79
53	F	55	Yes	T4NO	ADC/BAC	0	0	c34G>T pG12C	0	0	0	1.79	2.14	2.06
54	F	78	No	T2N0	ADC/BAC	c2239_2251delinsC, p747-751delinsP747	0	0	0	0	0	1.31	2.12	1.90
55	F	72	No	T2N2	ADC/BAC	L858R	COMPLEX INSDEL	0	0	0	0	1.65	1.81	1.72
57	M	85	Yes	T2N0	ADC	0	0	0	0	0	0	1.87	1.94	1.89
58	M	51	Yes	T2N0	ADC	0	c467G>C, pR156P	0	0	0	0	2.79	1.96	1.77
59	M	51	Yes	T2N0	ADC	0	0	c34G>T pG12C	0	0	0	2.10	1.39	1.92
60	M	71	Yes	T1NO	SCC	0	c-15-96del, Fs mutation	0	0	0	0	1.85	1.78	1.88
62	M	48	Yes	T4NO	ADC	0	C783-2A>G, SLPICEMUT	0	0	0	0	2.80	2.26	1.73
64	M	71	Yes	T3N1	ADC	0	c466-483del, FsI162H	0	0	0	0	2.86	2.12	0.69
65	M	49	Yes	T4NO	ADC/BAC	0	C817C>T, pR273C	0	0	0	0	2.33	2.24	2.02
66	M	56	Yes	T2N1	ADC	0	0	0	0	0	0	1.88	1.98	1.82
67	M	56	Yes	n.d.	ADC	0	0	c34G>T pG12C	0	0	0	3.25	2.84	1.93
68	M	58	Yes	T2N0	ADC	0	c818G>T, pR273L	c34G>T pG12C	0	0	0	3.26	1.86	1.44
69	M	72	Yes	T3N0	ADC	0	c610G>T, pE204X	0	0	0	0	3.17	1.55	2.19
70	M	71	Yes	T3N0		0	0	0	0	0	0	2.13	1.74	1.90
71	M	69	Yes	T3N1	ADC	0	C528C>G, pC176W	0	0	0	0	2.68	1.59	1.97
72	M	77	Yes	T3N2	SCC	0	c408DELA, FsQ136H	0	0	0	0	2.67	2.23	1.61
74	M	76	Yes	T2N0	SCC	0	c318G>C, pG105R	0	0	0	0	3.28	3.94	2.11
75	M	59	Yes	T3N2	SCC	0	c797delG, FsG266D	0	0	0	0	4.66	2.74	1.44
76	M	48	Yes	T3N0	ADC	0	0	c35G>T pG12V	0	0	0	2.32	2.92	1.90
78	M	67	Yes	T2N0	SCC	0	0	0	0	0	0	2.42	1.81	1.75
79	M	57	Yes	T2N0	ADC	0	c818G>T, pR273L	c34G>T pG12C	0	0	0	4.27	5.76	1.69
80	M	58	Yes	T1N2	ADC	0	0	c35G>A pG12D	0	0	0	6.36	1.66	1.47
82	M	62	Yes	T2N1	SCC	0	0	0	0	0	0	2.24	1.88	1.84
83	M	51	Yes	T3N0	ADC	0	C139-140DEL, pFsP47G c844C>T, pR282W	c34G>C pG12R	0	0	C521A>G, pM174R	1.87	2.38	1.98
84	M	67	Yes	T4N2	SCC	0	0	0	0	0	0	2.49	2.19	1.98
85	M	56	Yes	T2N0	ADC	0	C577C>G, pH193D	0	0	0	0	2.53	2.56	2.09
86	M	74	Yes	T1NO	SCC	0	0	0	0	0	0	2.19	2.04	1.84
87	M	52	Yes	T2N1	ADC	0	c595G>T, pG199X	0	0	0	c126-149del, pFsmutation	2.38	2.61	1.98
88	M	75	Yes	T4N1	SCC	0	C586C>T, pR196X	0	0	c1633G>A, pE545K	0	2.88	3.89	0.28
89	M	83	Yes	T4N1	SCC	0	c560-lG>T, SPLICEMUT	0	0	0	0	2.37	1.77	1.73
92	M	83	Yes	T2N0	SCC	0	0	0	0	0	0	1.31	0.66	1.86
93	M	50	No	T2N0	ADC	0	n.d.	0	0	0	0	3.81	1.44	1.72
94	M	73	Yes	T3N2	SCC	0	c785G>T, pG262V	0	0	0	0	1.40	1.53	1.31
95	M	64	Yes	T2N1	SCC	0	0	0	0	0	0	2.40	2.28	1.92
96	M	67	Yes	T2N2	SCC	0	c438G>A, pW146X	0	0	0	0	4.16	2.87	1.71
97	M	81	Yes	T4N2	ADC	0	0	0	0	0	0	7.44	2.68	1.64
98	M	57	Yes	T2N0	ADC	0	0	0	0	0	0	2.41	2.16	2.13
99	M	69	Yes	T2N1	SCC	0	C586C>T, pR196X	0	0	0	0	2.85	6.96	1.14
100	M	74	Yes	T3N2	SCC	0	0	0	0	0	0	2.69	2.42	1.73
101	M	51	No	T4N2	ADC	0	0	0	0	0	0	2.44	1.95	1.9
102	M	61	Yes	T2N0	SCC	0	c578A>G, pH193R	0	0	0	0	6.13	1.94	1.89
103	M	52	Yes	T2N0	ADC	0	0	0	0	0	0	2.42	1.34	1.87
104	M	75	Yes	T2N0	ADC	0	0	0	0	0	0	1.38	1.35	2.18
105	M	71	Yes	T2N0	ADC	0	C832DELC, pFsP278L	0	0	0	0	5.13	2.04	1.75
106	M	56	Yes	n.d.	SCC	0	C906DELG, pFsS303A	0	0	0	0	2.66	2.87	1.71
107	M	54	Yes	T1NO	ADC	0	c460G>A, pG154I	c34G>T pG12C	0	0	0	2.50	0.81	0.55
108	M	57	Yes	T3N1	SCC	P753L	C902DELC, pFsP301Q	0	0	0	0	6.91	1.55	1.09
109	M	52	Yes	T2N0	ADC	0	0	c34G>T pG12C	0	0	0	1.48	1.79	1.65
110	M	75	Yes	T1N1	SCC	0	0	0	0	0	0	3.11	1.71	1.97
111	M	65	Yes	T2N2	ADC	0	0	0	0	0	0	1.41	1.58	2.00
112	M	51	Yes	T1NO	ADC	0	0	c34G>T pG12C c35G>T pG12V	0	0	0	2.65	2.73	1.49
134	F	62	No	T2N0	ADC	L858R	c503A>T, pH168L	0	0	0	0	2.00	13.47	2.13
135	F	72	No	T1NO	BAC	c2237_2255delinsT, p746-752del insV746	0	0	0	0	0	1.31	1.16	0.46
143	F	55	Yes	T2N0	ADC/BAC	0	0	c34G>T pG12C	0	0	0	1.99	2.10	1.83
177	F	67	Yes	T2N0	ADC/BAC	0	0	c35G>A pG12D	0	0	0	2.00	1.94	1.71
246	F	44	Yes	T2N2	ADC	0	0	c34G>T pG12C	0	0	0	2.25	2.59	1.78

For quantitative PCR, an inferred copy  number ≥ 4 defines an amplification of target and an inferred copy number ≤ 0.6 defines  homozygous deletion. Tumors analyzed by Xba1 50000 SNPs array (Affymetrix) are  underlined.

### Relation between cyclin amplification and clinicopathological parameters

A significant association was found between *CCND1 *amplification and tobacco exposure (p = 0.023) and *TP53 *mutations were linked to *CCNE1 *(p = 0.006), or *CCND1 *(p = 0.048) amplifications (Table 4). One tumor showed simultaneous amplification of both cyclins.

**Table 4:** Statistical analysis: CCND1, CCNE1, CDKN2A associations with clinico-pathological  features and other gene alterations.

CDKN2A		CDKN2A presence of homozygous deletion n (%)	CDKN2A absence of homozygous deletion n (%)	p
Age		62	62,6	NS
Gender	Women	6 (12)	44 (88)	0,157
	Men	2 (4)	46 (96)	
Histology	ADC	7 (10)	61 (90)	0,483
	LCC	0 (0)	6 (100)	
	SCC	1 (4)	23 (96)	
Tobacco	Never-smoker	5 (20)	20 (80)	0,012
	Smoker	3 (4)	70 (96)	
	Mutated	5 (33)	10 (67)	
EGFR	Non-mutated	3 (4)	80 (96)	0,002
	Mutated	1 (5)	19 (95)	
KRAS	Non-mutated	7 (9)	71 (91)	0,563
	Mutated	1 (14)	6 (86)	
STK11	Non-mutated	7 (8)	84 (92)	0,46
	Mutated	3 (7)	40 (93)	
TP53	Non-mutated	5 (9)	50 (91)	0,704
AKT-mTOR activation*	Yes	7 (29)	17 (71)	<0,001
	No	1 (1)	73 (99)	

CCND1		Amplification n (%)	No amplification n (%)	P

Age		61,8	63	NS
Gender	Women	5 (10)	45 (90)	0,331
	Men	8 (17)	40 (83)	
Histology	ADC	6 (9)	62 (91)	0,079
	LCC	2 (33)	4 (67)	
	SCC	5 (21)	19 (71)	
Tobacco	Never-smoker	0	25 (100)	0,023
	Smoker	13 (18)	60 (82)	
EGFR	Mutated	1 (7)	14 (93)	0,331
	Non-mutated	12 (14)	71 (86)	
KRAS	Mutated	2 (10)	18 (90)	0,629
	Non-mutated	11 (14)	67 (86)	
STK11	Mutated	1 (1)	6 (99)	0,934
	Non-mutated	12 (13)	79 (87)	
TP53	Mutated	9 (21)	34 (79)	0,048
	Non-mutated	4 (7)	51 (83)	
AKT-mTOR activation*	Yes	2 (8)	22 (92)	0,412
	No	11 (15)	63 (85)	

CCNE1		Amplification n (%)	No-amplification n (%)	P

Age		67	62,5	NS
Gender	Women	2 (4)	47 (96)	0,371
	Men	4 (8)	45 (92)	
Histology	ADC	2 (3)	66 (97)	0,099
	LCC	1 (1)	5 (99)	
	SCC	3 (13)	21 (87)	
Tobacco	Never-smoker	1 (1)	24 (99)	0,518
	Smoker	5 (17)	68 (83)	
EGFR	Mutated	1 (1)	14 (99)	0,924
	Non-mutated	5 (7)	78 (93)	
KRAS	Mutated	1 (1)	19 (99)	0,814
	Non-mutated	5 (6)	73 (94)	
STK11	Mutated	0	7 (100)	0,483
	Non-mutated	6 (7)	85 (93)	
TP53	Mutated	6 (15)	37 (85)	0,006
	Non-mutated	0	55 (100)	
AKT-mTOR activation*	Yes	2 (8)	22 (82)	0,603
	No	4 (5)	70 (85)	

In this work we called PI3K-AKT-mTOR activated tumors, tumors with either EGFR,  PIK3CA, ERBB2 or STK11 mutation*.

### Relation between *CDKN2A *homozygous deletion and clinicopathological parameters

*CDKN2A *homozygous deletions were significantly associated with *EGFR *mutations (p = 0.002) and absence of tobacco exposure (p = 0.012). One tumor had *CDKN2A *homozygous deletion and *CCNE1 *amplification (Table 4). Three tumors from smokers had *CDKN2A *homozygous deletion, one had a *STK11 *and the other a *PIK3CA *mutation. It suggested that an activation of the EGFR/PI3K/AKT/mTOR pathway could be linked to *CDKN2A *homozygous deletion in lung cancer regardless of smoking status. All together, 8 tumors out of 9 with *CDKN2A *homozygous have EGFR/PI3K/AKT/mTOR pathway alterations.

## Discussion

In lung cancer, EGFR inhibitors have been shown to be efficient in tumors with activating mutations of the receptor. This molecular alteration defines a subgroup of patients with specific clinicopathological features. The most striking one is the fact that patients are mostly non-smokers. Understanding the carcinogenesis pathway that drives lung carcinogenesis in non-smokers is therefore of major interest. The present work represents the first comparative study of genome wide allelic imbalance between *EGFR *mutated lung ADC and *KRAS *mutated ADC. In our work, *EGFR *mutated tumors are from non-smoking women with ADC, the control group was selected among smoking women with ADC and without *EGFR *mutations. As most of them have *KRAS *mutations, we chose to consider for the SNP array only tumors with *KRAS *mutations in order to have a homogenous control group. Moreover, it was already suggested that *KRAS *and *EGFR *mutations were hallmarks of tobacco and non-tobacco induced lung carcinogenesis [17]. Different reports showed that SNP array technology provided the opportunity to assess DNA copy number and LOH through the entire genome [12,18-20]. The overall pattern of alterations seen in this study is reliable with previous lung ADC studies. As already noted, frequent chromosome gains are found at 1q, 5p, 7p/q, 8q and 14q and losses at 6q, 8p, 9p, 13q, 18q and 19p [11,20-22]. But new regions of homozygous deletion have been found in EGFR mutated tumors especially at 18q involving the DCC gene and a cluster of desmosomal proteins. New regions of focal amplification at 7p and 14q have also been delineated that encodes potential oncogenes [22].

Using this technology, we showed that genome wide allelic imbalance patterns are different in tumors from non-smokers when compared to that of smokers. Indeed, more LOH and amplified regions were found in the *EGFR *mutated group when considering either all regions > 5 SNPs or entire chromosome arm losses or amplifications. This fact has already been suggested in a previous work using microsatellite markers and showing that tumors in non-smokers had more alterations than that of smokers [23]. The mechanisms leading to this increased chromosomal instability is not yet understood. In this series *TP53 *mutations were equally distributed in both groups therefore the impact of TP53 on genome stability cannot be discussed. Other mechanism such as repair defects could be involved in increased chromosomal instability in non-smokers.

In parallel to DNA assays, RNA expression profiles also demonstrated differences between ADC from smokers when compared with non-smokers [24,25]. In a recent paper dealing with expression profiling of epidermal growth factor receptor/KRAS pathway activation in lung cancer two groups of ADC were individualized, one being a bronchial-type, the other an alveolar-type [26]. Unsupervised classification failed to detect any specific group of tumors that had *EGFR *mutations but they showed 26 genes preferentially expressed in *EGFR *mutated group. Among them, three genes *EGFR*, *PTK7 *and *HMOX2 *were found in our series located in focal regions of amplifications. Finally, a genetic classification of lung ADC by CGH array showed that *EGFR *mutated tumors could be individualized as a specific cluster [27]. All together, these results tend to prove that lung ADC in non-smokers forms a distinct disease at least at a molecular point of view. SNP arrays allow the identification of many loci with either LOH or amplification. Some of which may represent background alterations with no specific involvement in the carcinogenesis process. The identification of regions that are critical for tumor cell proliferation is of major importance. In order to go further in the identification of alterations linked to *EGFR *mutations we focused on genes implicated in cell cycle regulation and validated SNP array information on a large series of NSCLC. Previous genome analysis using SNP Arrays also showed *CDKN2A *homozygous deletions and *CCNE1 *amplifications in lung cancers [12]. We found a link between *CDKN2A *homozygous deletion and the non-smoking status as already suggested by Kraunz et al. [28]. Moreover, in our series *CDKN2A *deletion was associated with *EGFR *mutation and maybe with an activation of the EGFR/PI3K/AKT/mTOR transduction pathway. Indeed, 7 out of 8 tumors with *CDKN2A *homozygous deletion have *EGFR*, *STK11 *or *PIK3CA *mutations. Activation of this signal transduction pathway can lead to enhance transcription of cell cycle genes as *CCND1 *and it is not surprising that no cyclin amplification was observed in this group. For this group of tumors, it seems that proliferation is under the dependence of an activation of cell cycle through EGFR/PI3K/AKT/mTOR signaling and an inactivation of the cell cycle inhibitor *CDKN2A *preventing cells to down-regulate proliferation. The *CDKN2A *locus at 9p21 encodes two genes, one inhibits CDK4 mediated RB phosphorylation (*CDKN2A/p16*) and the other binds MDM2 leading to TP53 stabilization (*p14/ARF*). qPCR experiments reported here, analyzed homozygous deletions at 9p21 and therefore co-deletion of both genes. A recent paper showed that the p14/ARF protein was frequently down regulated in lung cancers with *EGFR *mutation or in tumors with *ERBB2 *mutations. As in our work, down regulation of the *CDKN2A/p14/ARF *locus could be linked to PI3K/AKT/mTOR activation as both *EGFR *and *ERBB2 *activate this pathway [29]. Although, regarding our results, it is very surprising that in their experiments, CDKN2A expression measured by immunohistochemistry remained positive when p14/ARF expression was extinguished. It suggested that *p14/ARF *could be down regulated independently of *CDKN2A *by mutation or promoter hypermethylation in *EGFR *mutated tumors. Promoter hypermethylation has indeed been shown to turn off *CDKN2A *locus in cancer but *CDKN2A *hypermethylation was linked to heavy smoking and squamous cell cancer which represents a different subgroup of tumors [30]. Here we suggest that *CDKN2A/p14/ARF *locus homozygous deletion could be an alternative mechanism to down regulate cell cycle inhibitors in ADC from non-smoking patients. In our series, Illumina GoldenGate Assay for methylation was used to quantify *CDKN2A *methylation, in the 24 tumors analyzed by SNP array. No difference in methylation status was found between EGFR, KRAS mutated tumors and non-tumor tissues (data not shown). Two other papers reported a negative correlation between *EGFR *mutation and *CDKN2A *methylation status in NSCLC [31,32]. Cyclin amplifications were in opposite related to tobacco and associated to *TP53 *mutations. In this case, cell cycle is activated through direct amplification of cyclins and *TP53 *inactivating mutations unable cells to repress proliferation. An association between *CCND1 *and *TP53 *was already suggested by protein expression analysis [33].

**Figure 2:**
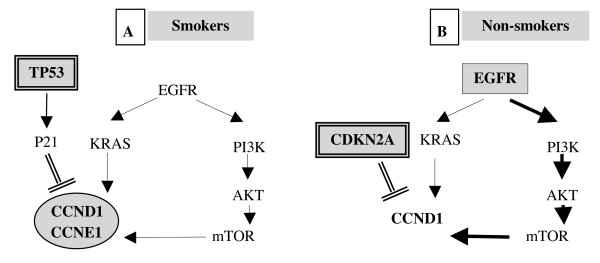
**Represents two possible models of oncogenic cooperation in smokers **(A) (Cyclin  amplification associated with TP53 mutations) and in non-smokers (B) (EGFR mutation  associated with CDKN2A homozygous deletion)

Although these models are not to generalize since other mechanisms such as mutations epigenetic alteration and protein overexpression can activate oncogene or inactivate tumor suppressor, our work enlightened two different carcinogenesis pathways in NSCLC. One is tobacco independent and driven by CDKN2A inactivation and EGFR mutations, the other is smoking dependent and driven by cyclin amplification and *TP53 *mutation (Figure 2).

## Conclusion

Although this work concerned a limited series of tumors, we focused on a comparative analysis and showed that patterns of genome wide genetic alterations are different between ADC with and without *EGFR *mutation. More alterations and a higher frequency of large alterations (entire chromosome arms deletion or amplification) were found in the *EGFR *mutated group suggesting that carcinogenesis pathways are different. Indeed, for a subset of tumors specific involvement of cell cycle genes were identified and an oncogenic cooperation between *EGFR *mutations and *CDKN2A *homozygous deletion was identified.

## Abbreviations

ADC: Adenocarcinoma; LCC: Large Cell Carcinoma; SCC: Squamous Cell Carcinoma; BAC: Bronchioloalveolar carcinoma.

## Competing interests

The authors declare that they have no competing interests.

## Authors' contributions

HB: Study design, analysis of the data, redaction of the manuscript. KP: quantitative PCR experiments, sequencing of target genes, analysis of the data. DLC: DNA extraction sequencing of target genes. CD: Study design, Pathological examination of the specimen. MT: quantitative PCR experiments. CH: sequencing of target genes. EF-G: Study design, collection of clinical data associated with tumor specimen. MR: Study design, collection of clinical data associated with tumor specimen. PD: Analysis of the data and redaction of the manuscript. PL-P: Study design, analysis of the data, redaction of the manuscript.

## Pre-publication history

The pre-publication history for this paper can be accessed here:



## Supplementary Material

Additional file 1 Provides a list of probes and primers used in the study for qPCR experiments. Click here for file

Additional file 2 Shows detailed statistical analysis using aCGH package. p was corrected for multiple testing situations. One region at 14q and three regions at 7p show meaningful differences between EGFR mutated and non-mutated tumors. Click here for file

Additional file 3 Shows regions of focal amplifications analyzed by Xba1 50000 SNPs array (Affymetrix) in EGFR mutated and KRAS mutated tumors independently of statistical differences. Genes, written in bold, have potential or obvious involvement in cell growth. For regions counting more than 10 genes only genes likely to have a role in carcinogenesis are given. Click here for file

Additional file 4 Shows the comparaison between the copy number estimated by the 50K SNP Array and qPCR. Click here for file
